# The development of immunosorbents for the treatment of systemic lupus erythematosus *via* hemoperfusion

**DOI:** 10.3389/fmed.2022.1035150

**Published:** 2023-01-04

**Authors:** Yameng Yu, Lailiang Ou

**Affiliations:** ^1^Key Laboratory of Bioactive Materials, Ministry of Education, College of Life Sciences, Nankai University, Tianjin, China; ^2^Beijing Key Laboratory of Digital Stomatology, NMPA Key Laboratory for Dental Materials, Department of Dental Materials, Peking University School and Hospital of Stomatology, National Center of Stomatology, National Clinical Research Center for Oral Diseases, National Engineering Laboratory for Digital, Material Technology of Stomatology, Research Center of Engineering and Technology for Computerized Dentistry Ministry of Health, Beijing, China

**Keywords:** immunosorbent, systemic lupus erythematosus, hemoperfusion, autoimmune disease, anti-double-stranded DNA antibody

## Abstract

Systemic lupus erythematosus (SLE) is an autoimmune disease (AID) that involves multiple organ systems and is characterized by elevated levels of autoantibodies (ANA) and immune complexes. The immunoadsorption technique uses an extracorporeal clearance process to remove pathogenic toxins from patients' blood and alleviate disease symptoms. An immunosorbent is a key component of the immunoadsorption system that determines therapeutic efficacy and safety. Immunosorbents are prepared by immobilizing antibodies, antigens, or ligands with specific physicochemical affinities on a supporting matrix. Immunosorbents and pathogenic toxins bind *via* affinity adsorption, which involves electrostatic interactions, hydrogen bonds, hydrophobic interactions, and van der Waals forces. Immunosorbents are classified on the basis of their interaction mechanism with toxins into three categories: non-selective, semi-selective, and highly selective. This review aimed to summarize the current status of various commercial immunosorbents that are used to treat SLE. Moreover, recent developments in immunosorbents have heightened the need for a brief discussion about specific ligands and a supporting matrix.

## 1. Introduction of immunoadsorption in the treatment of systemic lupus erythematosus

The immunoadsorption technique uses extracorporeal perfusion to selectively remove pathogenic toxins from patients with autoimmune diseases (AIDs) to purify the blood and alleviate disease symptoms (1). In contrast to traditional blood purification techniques, immunoadsorption is a novel treatment strategy based on antigen–antibody interactions. The immunoadsorption process is therefore unique to pathogenic toxins. It is important to note that immunosorbents play an essential role in determining therapeutic efficiency and safety as a result of direct contact with pathogenic toxins and blood components. A typical immunosorbent consists of a matrix and specific ligands, such as antigens, antibodies, or a ligand with specific physicochemical affinities (2, 3). Immunoadsorption is classified into two types: plasma perfusion and whole blood perfusion (4). In the former case, plasma must be separated before purification. Instead, blood is introduced directly into the adsorption column during whole blood perfusion, where pathogenic substances are selectively adsorbed by immunosorbents. Pure blood is reintroduced into a patient's body to achieve therapeutic goals.

Systemic lupus erythematosus (SLE) is an AID that can cause immune system dysfunction, loss of autoimmune tolerance, and abnormal activation of autoreactive lymphocytes, which can cause tissue and organ damage (5, 6). SLE is characterized by the production of various antibodies against deoxyribonucleic acid (DNA). The level of anti-double-stranded DNA (anti-dsDNA) antibodies is closely related to disease activity, which can cause pathology by directly inducing cell apoptosis. In addition to their direct effects, anti-dsDNA antibodies can also exert pathogenic effects in an indirect manner *via* circulating antigen–antibody complexes that form during the course of the disease (7, 8). Therefore, the removal of anti-dsDNA antibodies is widely recognized as an efficient treatment strategy that can benefit overall clinical outcomes (9–11). Plasma exchange can remove approximately 49–89% of autoantibodies (ANA) from the plasma of a patient but adverse effects limit the technique, resulting, for example, in a reduction of essential plasma components and in the induction of allergic reactions and viral contamination (12). In 1979, Terman et al. (13) published a case report involving DNA-activated carbon as an adsorbent for the removal of anti-dsDNA antibodies from the plasma of a patient with severe SLE. Since then, a wide range of immunosorbents containing specific ligands have been developed to remove anti-dsDNA antibodies from patients' blood *via* hemoperfusion (1–3). This study discusses the latest developments of various immunosorbents, from synthetic methodologies to technical specifications and therapeutic efficiencies.

## 2. Ligands for immunosorbents

**Figure 1:**
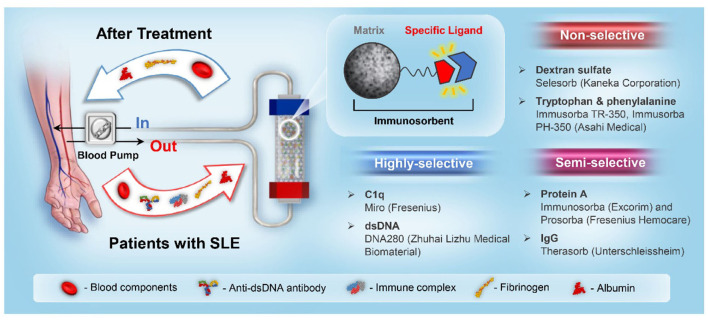
A schematic illustration of the SLE immunoadsorption system and immunosorbent classification.

The binding between immunosorbents and pathogenic toxins is based on affinity adsorption, which includes electrostatic interactions, hydrogen bonds, hydrophobic interactions, and van der Waals forces (14). According to the ligand and autoantibody binding principle, immunosorbents can be classified into three categories, as illustrated in Figure 1.

### 2.1. Dextran sulfate

Dextran sulfate is a specific ligand widely used in SLE immunosorbents and is an anionic dextran derivative with high binding affinity to anti-dsDNA antibodies *via* the cross-reactivity of repeating negatively charged units (15, 16). Liposorber LA40 (Kanegafuchi Chemical Industry) is a blood lipid purification system that contains cellulose gels grafted with dextran sulfate to selectively remove low-density lipoproteins (LDLs) from patients with familial hypercholesterolemia. Kinoshita et al. (17) reported that the Liposorber system could be highly effective in safely removing anti-dsDNA antibodies and immune complexes from the plasma of patients with SLE. In this study, 2 L of plasma were treated with a dextran sulfate gel column, and an anti-dsDNA antibody adsorption rate was achieved at up to 40%. Other biochemical indicators besides blood lipids, such as albumin, show no significant changes during immunoadsorption. However, anti-dsDNA antibodies have a lower adsorption capacity than LDL, total cholesterol, and triglycerides, which causes atherosclerosis. Based on those results, the Selesorb column was designed to achieve higher adsorption selectivity by increasing the amount of dextran sulfate immobilization and narrowing the cellulose matrix's pore diameter to match the size of anti-dsDNA antibodies. Anti-dsDNA antibody removal efficiency significantly increased as a result of the optimized pore diameter and ligand grafting density, and it was approximately two times as high as that of the Liposorber system (18).

The Selesorb system comprises two alternate columns, each containing 150 ml of porous cellulose beads grafted with dextran sulfate ligands and connected to a regenerating apheresis unit to recover adsorption efficiency during the treatment. Currently, the Selesorb system has been successfully utilized to treat patients with SLE. The relevant case series are summarized in Table 1. Suzuki et al. (21) reported a clinical trial of six patients with SLE who received Selesorb immunoadsorption. The levels of anti-dsDNA antibodies could be rapidly reduced after 2–4 treatment procedures for all subjects, and the mean adsorbing ratio of the antibodies was ~55.6% ± 4.6%. The symptoms improved in three subjects with proteinuria and four with lymphocytopenia after the apheresis procedure. In addition, Selesorb immunoadsorption could also remove anticardiolipin antibodies and immune complexes from the plasma of patients with SLE and improve the vascular changes and symptoms of arthralgia, rashes, and lymphocytopenia (20, 24). However, adverse events accompanied by treatment with Selesorb were also reported, including nausea, vomiting, hypotension, cardiopalmus, dizziness, chills, and thrombocytopenia. These symptoms were considered to be derived from hypovolemia or vasovagal reactions that are sometimes observed during extracorporeal therapies (18).

**Table 1:** A summary of the case reports with the application of Selesorb in the treatment of systemic lupus erythematosus (SLE).

**Subject** **number**	**Outcome** **parameters**	**Therapeutic** **effect**	**References**
*n =* 1	IgG, IgM immune complexes	Restored renal function	(19)
*n =* 1	IgG, IgM, IgA, C3	Improvement of skin lesions	(20)
*n =* 6	Proteinuria, anti-dsDNA antibody, CIC	Improvement of symptoms	(21)
*n =* 6	ACL, anti-dsDNA antibody	Decreased titers of pathogenic toxins	(22)
*n =* 19	SLEDAI, anti-dsDNA antibody	Decreased titers of pathogenic toxins	(23)

### 2.2. Tryptophan and phenylalanine

**Figure 2:**
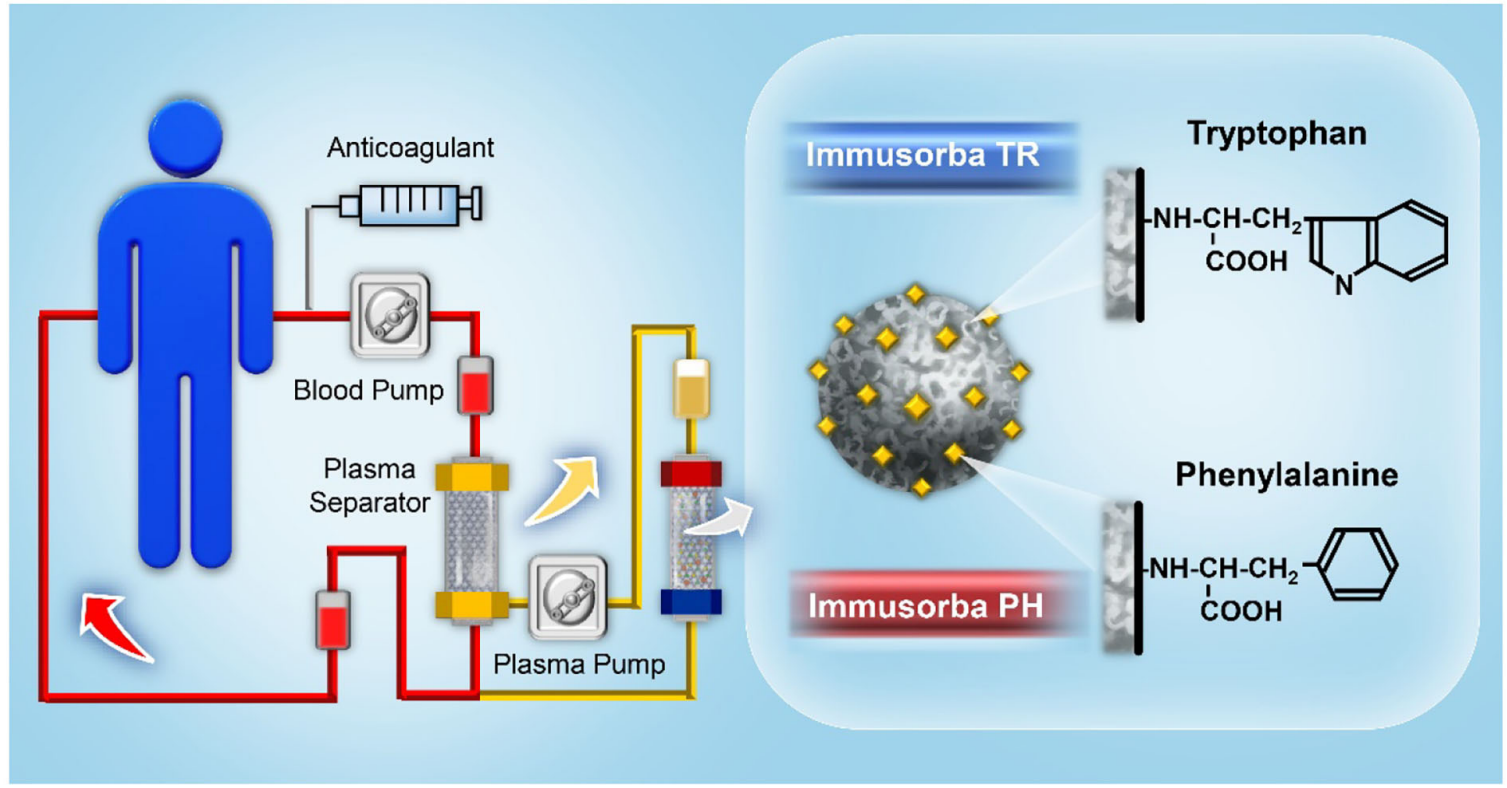
A schematic illustration of immunoadsorption using Immusorba TR and Immusorba PH.

Tryptophan and phenylalanine can be used as ligands to develop non-biological immunosorbents for the removal of pathogenic factors, such as anti-acetylcholine receptor antibodies, anti-ganglioside antibodies, and anti-DNA antibodies, *via* physicochemical interactions, including hydrophobic force or ionic interactions. Immusorba (Asahi Medical) was the first immunosorbent product bearing non-biological ligands; this system contains two types of columns, namely Immusorba TR and Immusorba PH, depending on the immobilized amino acid of the adsorbent (25, 26). Tryptophan is used as a ligand for Immusorba TR, and phenylalanine is used as a ligand for Immusorba PH. Porous poly(vinyl alcohol), a supporting matrix, was treated with epichlorohydrin to activate the matrix, followed by covalent immobilization of tryptophan or phenylalanine to prepare adsorbents. A schematic illustration of immunoadsorption using Immusorba TR and Immusorba PH is shown in Figure 2.

An *in vitro* study demonstrated that both Immusorba TR-350 and Immusorba PH-350 possess high removal efficiencies of anti-dsDNA antibodies and circulating immune complexes (CICs) from the plasma of patients with SLE. The reduction rate of anti-dsDNA antibodies was ~65% ± 15% on Immusorba TR-350 and 75% ± 14% on Immusorba PH-350. The reduction rate of CIC was ~75% ± 2% on Immusorba TR-350 and 74% ± 3% on Immusorba PH-350 (27, 28). Avenhaus et al. (28) evaluated the adsorption efficiency and underlying binding mechanism of Immusorba in an *in vitro* miniature mode to simulate the immunoadsorption procedure. The average reduction rate of anti-dsDNA antibodies was significantly higher than that of both immunoglobin G (IgG) and total protein. Hydrophobic interactions served as the predominant force in the binding between the ligand phenylalanine and pathogenic antibodies. Sugimoto et al. (29) reported a clinical trial of six patients with lupus nephritis (LN) associated with proteinuria and abnormal sedimentation on urinalysis. Those patients were treated by immunoadsorption plasmapheresis using Immusorba PH-350. The levels of anti-DNA antibodies decreased significantly after apheresis. The levels of urinary protein, immune complexes, and other pathogenic substances were also reduced, indicating the treatment efficiency of LN.

Similar to the dextran sulfate gel column, few studies showed that an immunosorbent bearing tryptophan or phenylalanine is applicable to more than one disease due to its broad-spectrum adsorption properties (30, 31). Non-selective adsorption might induce non-ideal consumption of fibrinogen. *In vitro* and *ex vivo* biocompatibility studies showed that fibrinogen concentration decreased remarkably to around 50% (32). Therefore, to avoid the consumption of beneficial serum components during apheresis, immunosorbents with highly specific ligands are required to provide superior therapeutic efficacy in the treatment of SLE.

### 2.3. Staphylococcal protein A

**Figure 3:**
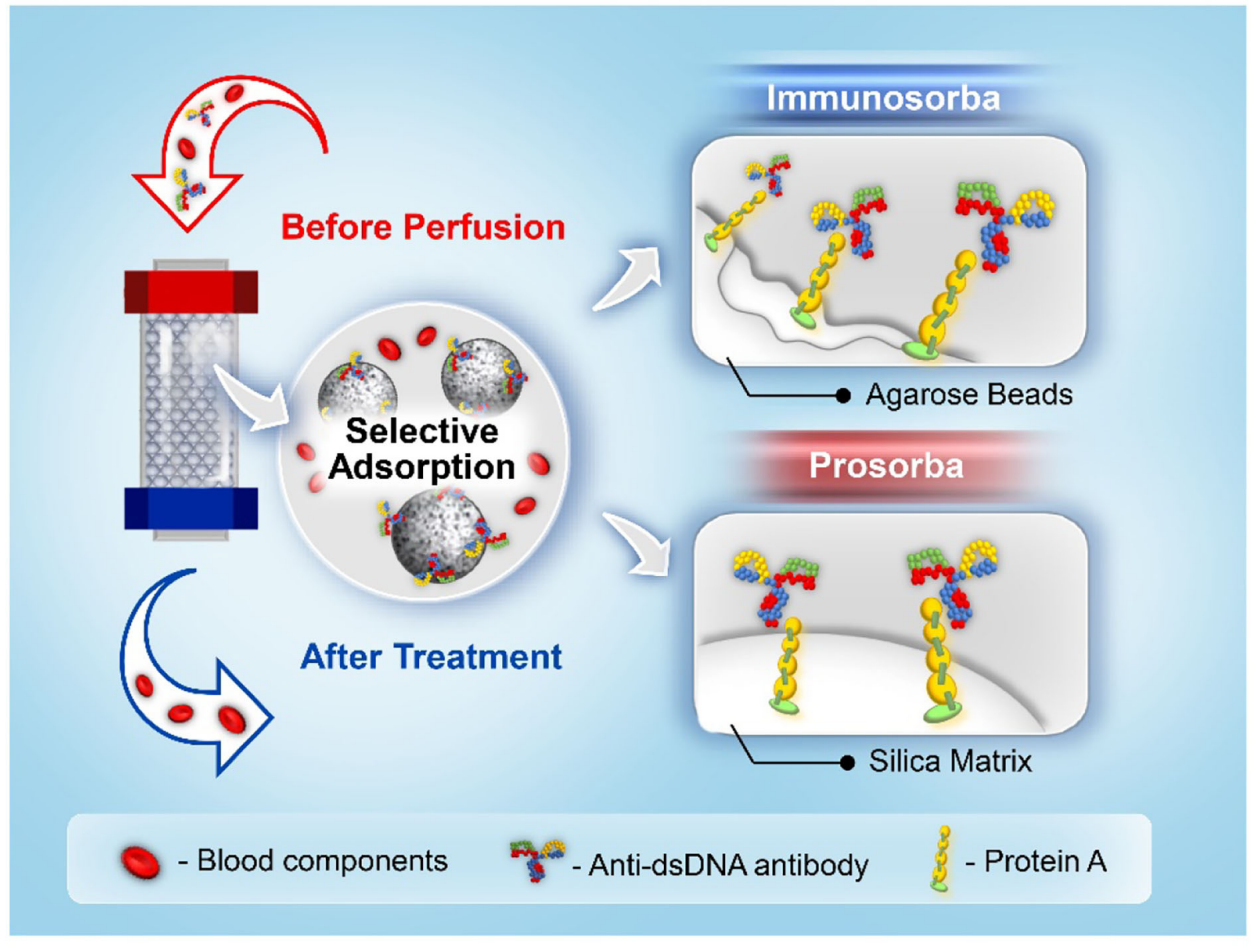
A schematic illustration of protein A Immunosorba and Prosorba.

*Staphylococcus aureus* protein A can selectively bind to the heavy chain within the fragment crystallizable (Fc) region of antibodies, particularly those of the IgG class. To date, two types of immunosorbents that use protein A as a specific ligand have been approved for clinical use by the US Food and Drug Administration, namely Immunosorba (Excorim) and Prosorba (Fresenius Hemocare) (33, 34). As shown in Figure 3, the supporting matrix is the crucial difference between the two immunosorbents. Protein A is immobilized onto agarose beads by cyanogen bromide activation, whereas protein A is coupled to a silica matrix by Prosorba.

The Immunosorba system consists of two adsorption columns and an elution monitor (35). During the treatment, both columns are used alternately in the adsorption–elution procedure, which allows the previously saturated column to be regenerated for a new perfusion cycle. Due to the high regeneration efficiency (more than 20 times) and large plasma operating volume, the levels of ANA in the patient's blood can be considerably decreased with the treatment of the Immunosorba protein A system. As the binding affinity between protein A and immunoglobulins is much higher than that of tryptophan, phenylalanine, or dextran sulfate, Immunosorba exhibited superior adsorption efficiency of protein A for CIC (73 ± 0%) and anti-dsDNA antibodies (83 ± 2%) from the plasma of patients with SLE compared to non-selective adsorbents (27). The Immunosorba protein A system can be used to remove total IgG, ANA, and CIC with concomitant amelioration of inflammation. A case report of eight patients with life-threatening or severe therapy-resistant SLE revealed that disease remission was achieved in seven patients with immunoadsorption (36). After the treatment, lupus activity measured by the systemic lupus activity measure (SLAM) index decreased from 23.8 ± 4.2 to 7.9 ± 4.3, and the best treatment efficacy was achieved through daily immunoadsorption.

Unlike the Immunosorba system, the Prosorba system only possesses one disposable column containing 200-mg protein A-immobilized silica beads (125 g). The maximum operating volume of the plasma in the Prosorba system is restricted to 2,000 ml, resulting in a relatively lower amount of immunoglobulins and a relatively lower removal capacity of CIC than that of Immunosorba (37). However, it has been reported that the leakage of protein A from the adsorbent during the treatment may have adverse effects in clinical applications (38). Protein A is a B-cell superantigen that evolved in Staphylococcus to weaken the host antibody-mediated defenses; thereby, exposure to protein A during the treatment might induce an immunosuppressive effect (39). The Food and Drug Administration (FDA) has discontinued the application of Prosorba since 2006 due to the aforementioned adverse effects in clinical use, and the Prosorba system has not been available (40).

### 2.4. Immunoglobin G

Immunoglobin G is recognized as a four-chain monomer, accounting for 75% of the total amount of serum immunoglobin, and is the most essential component of antibodies in serum and extracellular fluids. IgG-based immunosorbents were developed, namely Therasorb, which utilizes polyclonal sheep antihuman IgG antibodies covalently coupled with cellulose beads to remove immune complexes and pathogenic antibodies through highly specific antigen–antibody interactions (41).

The Ig-Therasorb system comprises two columns containing 150 ml of the adsorbent and can be regenerated using glycine buffer *via* an automatic adsorption–desorption apparatus. It has been widely reported that Ig-Therasorb immunoadsorption can reduce the anti-dsDNA antibody levels, disease activity, and proteinuria. The results are summarized in Table 2. Gaubitz et al. (43) reported a randomized trial to compare the efficiency of Ig-Therasorb and Immusorba PH-350 in treating patients with SLE. Approximately 20 patients suffering from moderate or severe SLE were randomized to receive hemoperfusion with either Ig-Therasorb or Immusorba PH-350. Both immunoadsorption systems showed satisfactory removal efficiencies of pathogenic antibodies without causing any adverse side effects. The removal rates of anti-ds-DNA antibodies were ~61.0 ± 10.8% and 50.8 ± 6.6% for Ig-Therasorb and Immusorba PH-350, respectively. The higher antibody binding efficacy might be attributable to the particular mode of action of IgG compared to the hydrophobic interactions of phenylalanine. The clinical outcomes of both systems were similar after 1 month of treatment. Still, the number of non-responders was higher in Immusorba PH-350 than in Ig-Therasorb due to the interindividual variability, different indications, and disease duration and severity of the small subject group. Nevertheless, some studies reported the occurrence of infections that may prevent the further application of this system (42, 45).

**Table 2:** Summary of the case reports involving the application of Ig-Therasorb in treating SLE.

**Subject** **number**	**Outcome parameters**	**Therapeutic effect**	**References**
*n =* 16	Anti-dsDNA antibody	Reduced proteinuria and disease activity	(42)
*n =* 10	Anti-dsDNA antibody	Decreased SLAM scores and anti-dsDNA antibody level	(43)
*n =* 1	Anti-dsDNA antibody	Decreased proteinuria and anti-dsDNA antibody level	(44)
*n =* 16	Anti-dsDNA antibody	Decreased disease activity and anti-dsDNA antibody level	(45)
*n =* 2	-	Safe and well tolerated in pregnant women	(46)

### 2.5. C1q

C1q is a component of the first complement component C1, with a molecular weight of 410 kDa. The C1q molecule is a heterohexamer composed of six subunits, each of which is composed of three polypeptide chains. Those peptide chains are connected by disulfide bonds to form a collagen-like structure, which can provide specific binding forces with CIC through the globular C-terminus. Gazitt et al. (47) developed a C1q-based immunosorbent that uses agarose polyacrolein microsphere beads as the supporting matrix to remove CIC in patients with AIDs based on this principle.

Miro (Fresenius) is a single-use immunosorbent containing 300 ml of C1q-immobilized porous polymer beads. Pfueller et al. (48) reported the results of a case report involving eight patients with SLE who received C1q immunoadsorption. The results demonstrated that the reduction of the European Consensus Lupus Activity Measurement score was observed in seven out of eight patients, the decrease in CIC-IgG was observed in five of eight patients, and the reduction of C1q-bearing immune complexes was observed in seven of eight patients. Furthermore, the inflammation parameters, such as the erythrocyte sedimentation rate, C-reactive protein, and the fibrinogen levels, were also decreased for all subjects. The satisfactory treatment efficacy of the C1q immunoadsorption system may be attributable to the multifunctional interactions between C1q and the pathogenic factors, such as CIC, anti-C1q ANA, and inflammatory proteins.

### 2.6. Double-stranded DNA

Double-stranded DNA is a DNA molecule composed of two single strands of DNA joined by the complementary action of bases. In 1979, Terman et al. (13) in a case report (13), demonstrated the use of DNA collodion charcoal immunosorbent to treat a 29-year-old woman with severe LN. Calf thymus DNA was immobilized in collodion membranes that were adhered to small charcoal particles as the adsorbents. Immune complexes and ssDNA antibodies could be substantially reduced by extracorporeal immunoadsorption. However, hemoperfusion with activated charcoal may result in adverse effects, such as particle embolism, and damage to blood cells, which can be alleviated by modifying collodion-activated charcoal with albumin to enhance blood compatibility (49).

**Figure 4:**
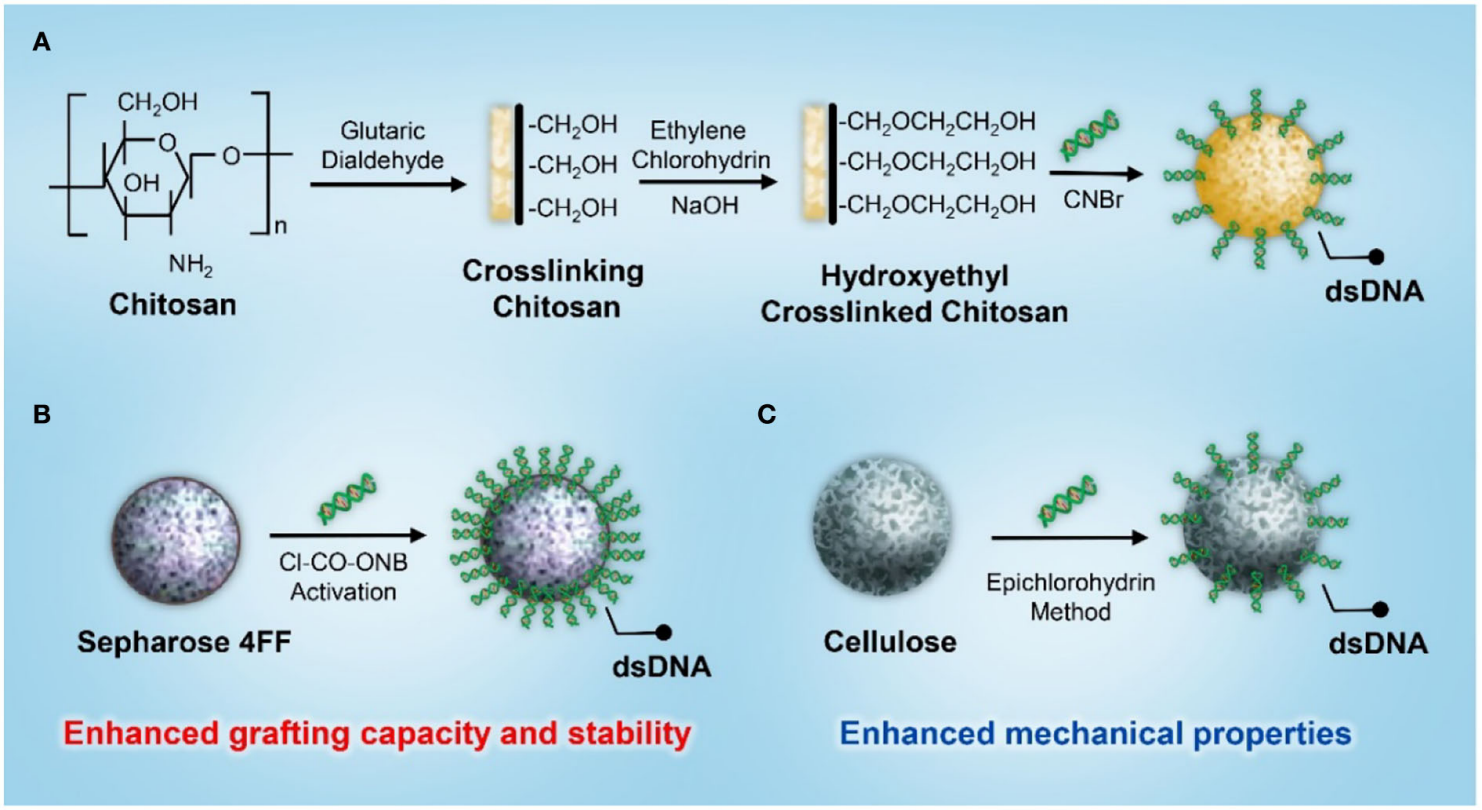
A schematic illustration of the synthesis of a dsDNA-based immunosorbent using **(A)** chitosan, **(B)** Sepharose 4FF, and **(C)** cellulose as the supporting matrix.

In addition to activated charcoal, various porous supporting matrices based on chitosan, Sepharose, and cellulose have been developed for DNA immobilization. Yu et al. (50) developed a DNA-based immunosorbent with the immobilization of calf thymus DNA onto hydroxyethyl-crosslinked chitosan beads *via* the activation of cyanogen bromide (Figure 4A). The adsorbent could effectively reduce the levels of anti-DNA antibodies in the serum of patients with SLE by 65.33% and could be regenerated for three adsorption cycles by glycine–hydrochloride (HCL). However, the preservation methods affected the performance of antibody adsorption. The adsorption capacity was found to be higher in dry conditions than in wet conditions. The decreased adsorption capacity might be attributed to the decomposition or hydrolyzation of the immobilized DNA under wet conditions.

To improve the grafting capacity and stability while reducing the leakage of the coupled DNA molecules, Kong et al. (51) developed an efficient strategy for DNA immobilization using Sepharose 4FF as the supporting matrix and 5-norbornene-2,3-dicarboximido carbonochloridate (Cl-CO-ONB) for matrix activation (Figure 4B). The coupling efficiency was investigated by varying pH, temperature, reaction time, DNA concentration, and the activation level of the supporting matrix. The results showed that the maximum amount of immobilized DNA was approximately 1.0 mg/ml under an optimal reaction condition, and the amount of DNA immobilization increased with increasing concentration and activation levels. The adsorbent exhibited excellent adsorption capacity and efficacy for anti-DNA antibodies. The removal rate of anti-DNA antibodies from the plasma of patients with SLE was approximately 80–90%. In addition, the adsorption rate for both normal IgG and total protein was < 15%, indicating satisfactory adsorption selectivity against beneficial blood components. However, Sepharose's poor flow through parameter and narrowed column type adaptation restricted its further application in SLE hemoperfusion, so different supporting matrices and coupling strategies were investigated in the development of a DNA-based immunosorbent. Based on its superior mechanical properties and the capacity to fulfill a relatively large column, cellulose was applied as the supporting matrix for DNA immobilization (Figure 4C). A cellulose-based DNA immunosorbent exhibited excellent adsorption capacity, and the antibody removal rate was ~80% at an adsorption ratio of 20:1 (plasma/adsorbent, v/v) (52).

In 1988, Yang et al. (53) reported a study on a new DNA immune adsorbent for hemoperfusion in SLE therapy. In this study, a patient with SLE who had high levels of anti-DNA antibodies and immune complexes was successfully treated with whole blood perfusion using a DNA immunosorbent. Calf thymus DNA was applied as the specific ligand and immobilized onto carbonized resin beads with an immobilization amount of 98–98.5%, and no leakage was detected during the perfusion process. Anti-DNA antibody levels decreased sharply from 56.34 to 0.8% after 2.5 h of hemoperfusion without significant clinical complications. Yu et al. (54) reported that 30 cases of clinical trials were performed in 12 hospitals in China using type I (DNA immobilized on the carbonized resin) and type II (DNA immobilized on cellulose) adsorbents for immunoadsorption. The levels of anti-DNA antibodies could be reduced by ~40–70% after whole blood perfusion; almost all the symptoms could be relieved, and some subjects were free from medicine administration. Type II adsorbents possessed a higher DNA immobilization capacity (0.6 mg/ml) than type I adsorbents (0.4 mg/ml). *In vitro* static adsorption experiments revealed that anti-DNA antibody removal efficiency on type II adsorbents was approximately 60%, which was significantly higher than that on type I adsorbents (30%). The high adsorption capacity might be attributable to the introduction of 1,6-hexamethylene diamine, which is the space arm during the preparation of adsorbents so that the steric hindrance effect could be effectively mitigated in the DNA immobilization and antibody adsorption processes.

In 2015, Xu et al. (55) conducted a study to evaluate the therapeutic efficacy of DNA280 (Zhuhai Lizhu Medical Biomaterial), a DNA immunoadsorption system comprising an enveloped carbonized resin as a matrix and purified DNA molecule fragments as ligands. This study involved 57 patients with severe SLE who received immunoadsorption between January 2007 and December 2013. The levels of ANA and anti-ds-DNA antibodies in such patients could be significantly reduced *via* immunoadsorption. The levels of immunological parameters, such as erythrocyte sedimentation rate, C-reactive protein, serum creatinine, and urine protein, were also significantly decreased compared to those before the treatment, and no severe adverse effects were observed during or after the treatment. However, the fabrication procedure of DNA280 was relatively complicated due to the cumbersome processes of matrix preparation, DNA extraction, and purification. Consequently, cost efficiency for such immunosorbents should be considered and improved.

## 3. Recent developments in immunosorbents for SLE

### 3.1. Advances in the supporting matrix

**Figure 5:**
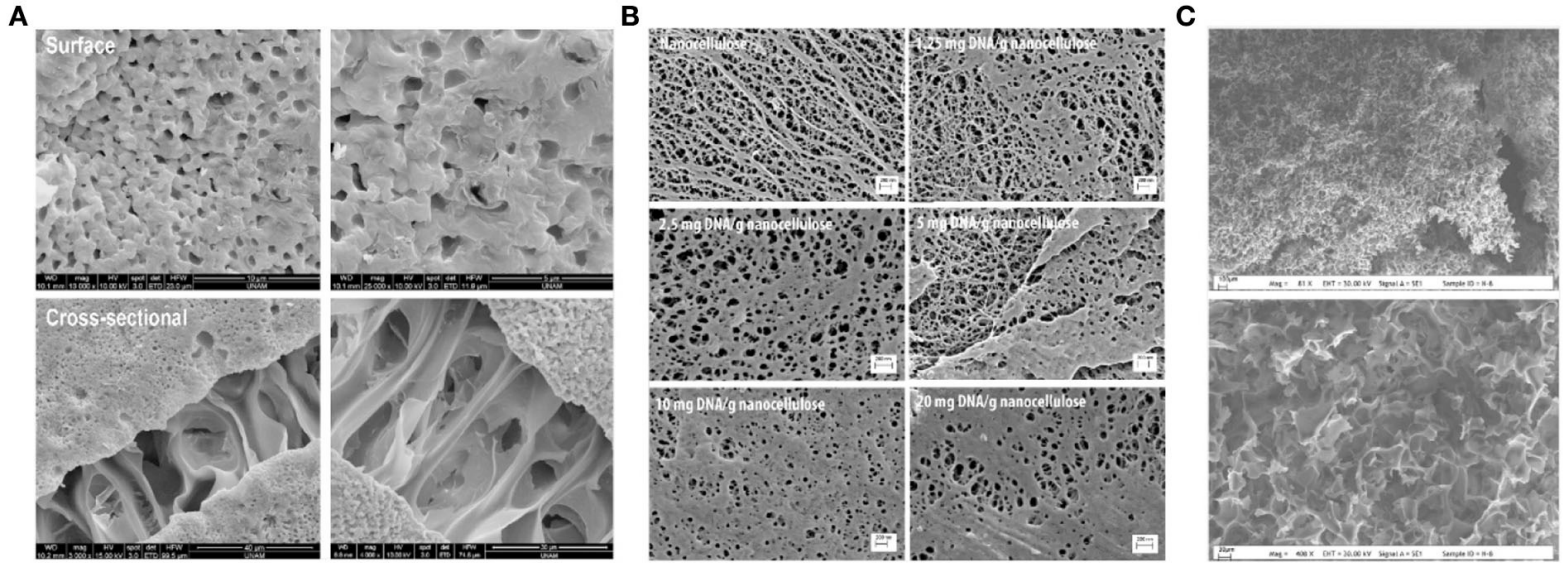
Scanning electron microscope images of **(A)** PHEMAAL membranes (57), **(B)** nanocellulose samples with indicated amounts of immobilized DNA (58), and **(C)** PHEMA cryogel (59).

Although immunoadsorption has been recognized as an effective therapeutic strategy for SLE, there are some non-negligible issues with the treatment procedure. For example, the compressed pressure of adsorbents in the column caused a reduced flow rate, especially when dealing with highly viscous blood samples (56, 57). Porous membranes are therefore considered alternative adsorbent geometries that can be applied under low-pressure conditions. Poly (2-hydroxyethyl methacrylate) (PHEMA) porous membrane possesses a large specific surface area, high chemical, biological, and mechanical stabilities, strong hydrophilicity, and antifouling properties, making it an ideal support matrix for the preparation of an immunosorbent for SLE. Uzun et al. (57) immobilized DNA onto a PHEMA-based microporous membrane (PHEMAAL-DNA) for the selective removal of anti-dsDNA antibodies from the plasma of patients with SLE. To further improve the compatibility of the blood membrane, N-methacryloyl-L-alanine (MAAL) was introduced as a monomer to copolymerize with HEMA to form the PHEMAAL membrane. As shown in Figure 5A, the membrane surface seems to be rough and heterogeneous, with largely interconnected pores of approximately 5–10 μm. The microporous structure and higher inner surface area could effectively decrease diffusional resistance and facilitate the mass transfer of anti-dsDNA antibodies. Anti-dsDNA antibody adsorption capacity was approximately 68 × 10^3^ IU/g. The levels of anti-dsDNA antibodies can be decreased from their initial value of 875–144 IU/ml. The adsorption membrane also exhibited excellent blood compatibility due to the incorporation of hydrophilic MAAL, which could effectively reduce blood cell adhesion and non-specific protein adsorption.

Cellulose materials have been widely applied in immunoadsorption due to their desired mechanical strength, physicochemical stability, and biocompatibility. However, conventional cellulose-based immunosorbents are limited in their ability to graft ligands owing to their low porosity and surface area. As compared to conventional cellulose materials, nanocellulose exhibits a surface area that is nearly two orders of magnitude greater than conventional cellulose materials. Based on this unique property, Xu et al. (58) prepared a DNA-immobilized immunosorbent using the nanocellulose membrane as the supporting matrix. As shown in Figure 5B, the non-coated nanocellulose exhibited a fibrous structure, and with an increased amount of immobilized DNA, new structures progressively emerged in the form of patches that covered the voids between the nanofibers. These patches become denser at the highest degree of DNA binding, which causes them to clog the pore structure. The nanocellulose-based DNA membrane exhibited a high affinity for binding with anti-ds-DNA IgG *in vitro*. The binding capacity was quantitatively dependent on the number of immobilized DNA ligands on the membrane.

Apart from the membrane, macroporous cryogel was applied as the supporting matrix to prepare an immunosorbent to facilitate blood cells passing through, rather than being blocked by, the pores. Özgür (59) modified PHEMA cryogel with polyethyleneimine (PEI) and DNA for the removal of anti-dsDNA antibodies from the plasma of patients with SLE. The cryogel-based adsorbent possessed a high porosity, approximately 67.5%, and interconnected macropores of 10–200 μm (Figure 5C). As a result of its macroporosity and interconnected pore structure, the adsorbent had a meager flow resistance. The maximum adsorption capacity of the anti-dsDNA antibody was approximately 70 × 10^3^ IU/g. The levels of an anti-dsDNA antibody in SLE plasma could be decreased from 780 to 80 IU/ml after adsorption.

### 3.2. Adsorbents using 4-mercaptoethylpyridine as a ligand

Applications of immunosorbents that use proteins or antibodies as ligands are limited because of their high cost and lack of stability. 4-Mercaptoethylpyridine (MEP, MW 139 Da) is a synthetic compound with desirable hydrophobicity that can be used to capture and purify antibodies from complex feedstock *via* hydrophobic interactions. Ren et al. (60) developed an MEP-grafted Sepharose gel to remove ANA from the serum of patients with AIDs. The MEP grafting density was optimized to achieve the maximum binding capacity for anti-dsDNA antibodies. The MEP Sepharose gel with a ligand density of 98.9 μmol/ml could remove 80% of the anti-dsDNA antibodies. Moreover, MEP-grafted Sepharose gel exhibited a lower degree of individual differences compared to Protein A-Sepharose. Out of the 14 serum samples derived from patients with SLE, 11 samples had markedly reduced antinuclear antibody titers. Albumin, fibrinogen, and other plasma components would not be virtually affected by the immunosorbent.

## 4. Conclusion

In the last three decades, immunoadsorption has been extensively used for treating patients with SLE who are refractory to conventional therapies. Unlike plasma exchange, immunoadsorption can effectively remove pathogenic antibodies and immune complexes without altering the levels of beneficial blood components. Immunosorbents containing non-selective or semi-selective ligands, such as dextran sulfate, phenylalanine, tryptophan, protein A, and IgG, have been commercially used over the past few decades. Among these, immunoadsorption columns, such as Ig-Therasorb and protein A Immunosorba, can remove disease-specific antibodies with a high affinity, making them valuable treatment options for SLE. Selesorb and Immusorba are examples of low-affinity columns that exhibit lower levels of efficacy. Ligands with higher selectivity are still being investigated, such as C1q and dsDNA. A non-selective or sim-selective immunosorbent may reduce the levels of beneficial plasma components and proteins after purification. A highly selective immunosorbent has the potential to bind to pathogenic antibodies with high specificity. However, further investigation is warranted to confirm its safety and efficacy. Recently, several studies focused on optimizing the supporting matrix and developing novel specific ligands to reduce treatment costs further and improve therapeutic efficacy and safety.

## Author contributions

YY: writing–original draft. LO: writing–review and editing. All authors contributed to the article and approved the submitted version.
